# Supplementary material for: Early Transport Patterns and Influencing Factors of Different Stocks of Uroteuthis edulis in the East China Sea
Source: Animals (Basel). 2024 Mar 19;14(6):941. doi: 10.3390/ani14060941 (PMC10967503; doi:10.3390/ani14060941)
Supplement: Supplementary file 1 [file animals-14-00941-s001.zip › animals-2831731-supplementary.pdf]

Table S1. Sampling information of *U. edulis* from the northern East China Sea. M represents males, and F represents females.

Sampling Dates	Locations	Quantity	Mantle Length/mm	Body Weight/g	Age/d	F:M
September 2018	127°E, 30°30′N	102	62-198	37-259	164-239	1.2:1
October 2018	127°E, 31°N	78	74-223	52-304	169-244	0.4:1
November 2018	126°30′E, 31°N	30	95-170	64-182	182-220	1:1
December 2018	127°E, 31°30′N	36	86-222	63-293	177-242	0.8:1
January 2019	127°E, 31°N	44	60-227	42-284	150-260	0.3:1
September 2019	124°E, 29°N	80	95-172	51-212	178-234	0.4:1
October 2019	127°30′E, 31°30′N	50	96-206	51-312	176-258	1.3:1
November 2019	127°E, 31°N	40	45-242	47-356	174-254	0.4:1
December 2019	126°30′E, 31°N	8	174-231	255-295	236-267	1:1
January 2020	126°30′E, 31°30′N	46	56-284	35-325	149-301	0.7:1
September 2020	125°30′E, 29°N	80	69-191	50-161	180-243	0.6:1
October 2020	127°E, 30°30′N	80	55-203	42-248	161-261	0.4:1
November 2020	127°E, 31°N	70	84-215	51-274	181-269	0.6:1
December 2020	127°E, 31°N	29	82-221	56-208	180-264	1.3:1

Table S2. Clustering of statolith trace elements of *U. edulis* based on MRTs. D is the distance from the statolith core to the edge of the longitudinal section.

Stock	Distance μm				
	Embryonic	Larval	Juvenile	Sub-adult	Adult
Spring	D≤60	60<D≤120	120<D≤240	240<D≤360	D>360
Summer	D≤60	60<D≤120	120<D≤180	180<D≤360	D>360

Figure captions

Figure S1. Particle tracking release area of *U. edulis* in the East China Sea. The sampling stations are represented by 2018, 2019, and 2020. Areas A and B showed the locations of particle tracking released from the spring and summer stock, respectively.

Figure S2. Relationship between cumulative daily increment and age in the statolith. S1, S2, S3, S4, and S5 represent the embryonic, larval, juvenile, sub-adult, and adult stages, respectively. a and b represent the spring and summer stocks, respectively. Lines represent linear regression predictions.

Figure S3. Particle tracking trajectories of the April group (spring stock) for 120 days in different sampling years. Red areas show the release locations of particle tracking. Blue lines show tracer trajectories for each particle. The numbers above the figures represent the release dates of particles.

Figure S4. Particle tracking trajectories of the May group (spring stock) for 120 days in different sampling years. Red areas show the release locations of particle tracking. Blue lines show tracer trajectories for each particle. The numbers above the figures represent the release dates of particles.

Figure S5. Particle tracking trajectories of the July group (summer stock) for 120 days in different sampling years. Red areas show the release locations of particle tracking. Blue lines show tracer trajectories for each particle. The numbers above the figures represent the release dates of particles.

Figure S6. Distribution of main currents and branches in the East China Sea.

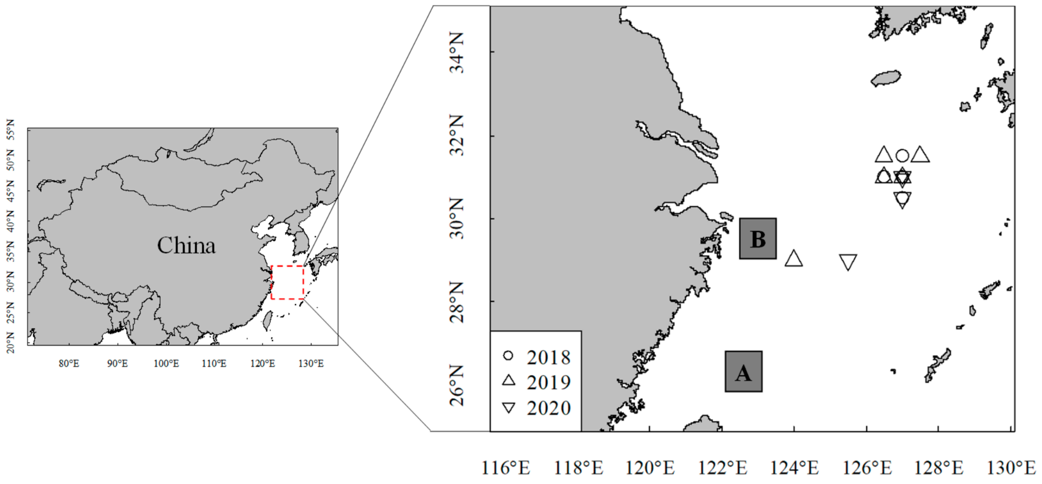
China
2018
2019
2020
A
B
34°N
32°N
30°N
28°N
26°N
116°E
118°E
120°E
122°E
124°E
126°E
128°E
130°E
80°E
90°E
100°E
110°E
120°E
130°E

Figure S1.

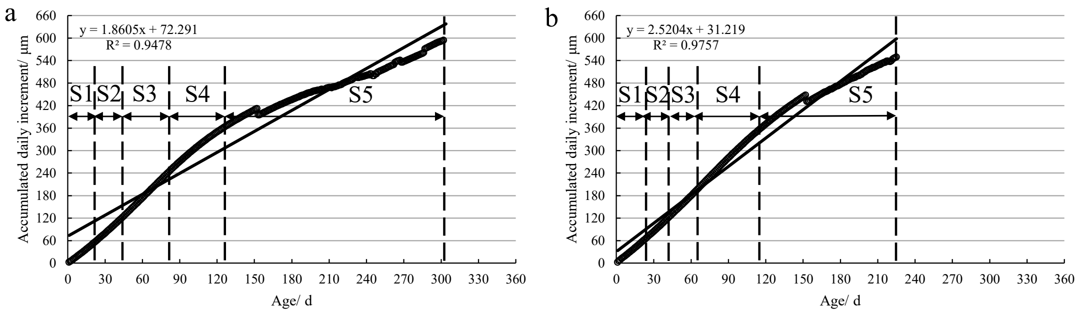

a
y = 1.8605x + 72.291
R² = 0.9478
S1 S2 S3 S4 S5
Accumulated daily increment/ µm
Age/ d
b
y = 2.5204x + 31.219
R² = 0.9757
S1 S2 S3 S4 S5
Accumulated daily increment/ µm
Age/ d

Figure S2.

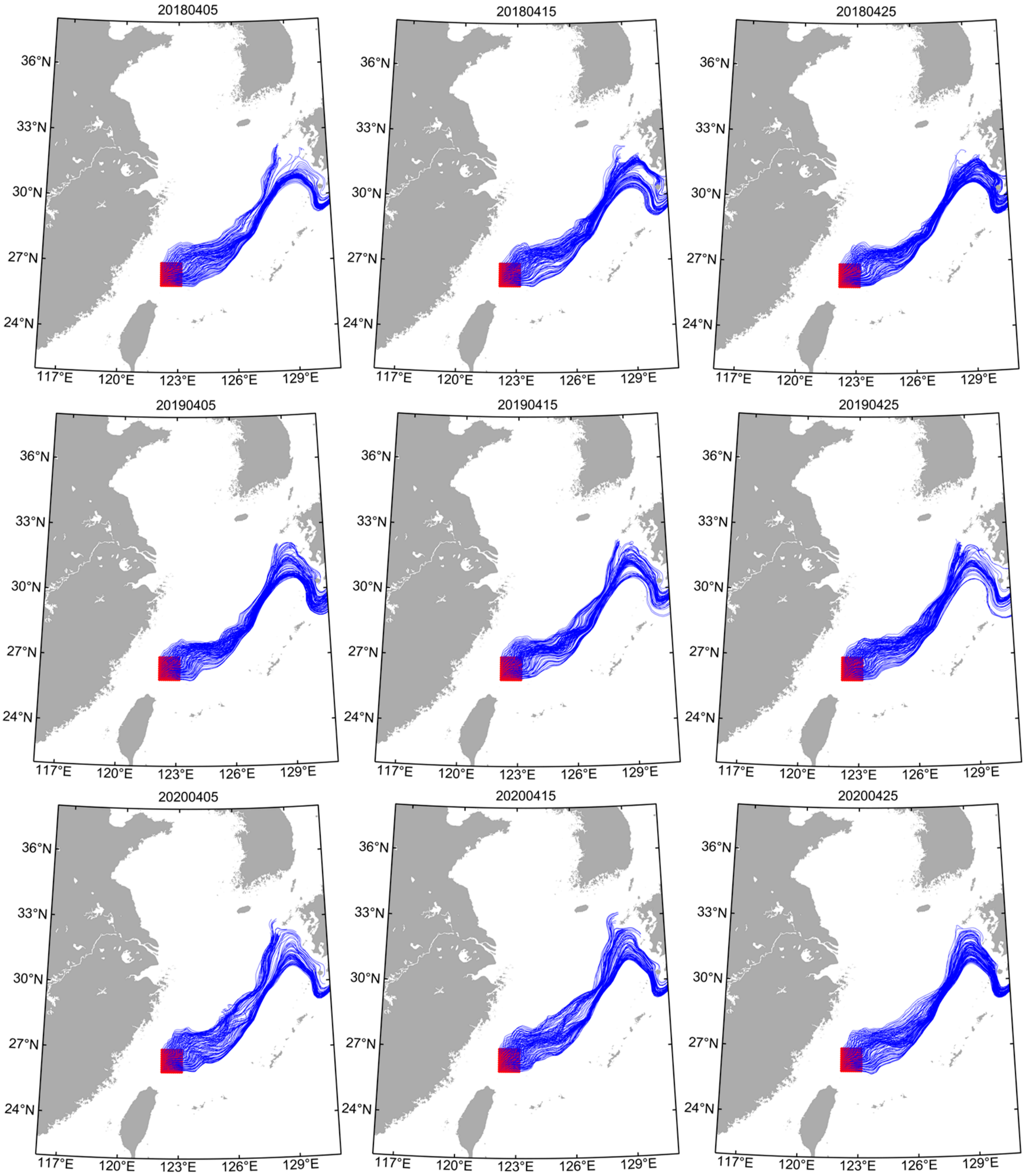

20180405
20180415
20180425
20190405
20190415
20190425
20200405
20200415
20200425
36°N
33°N
30°N
27°N
24°N
117°E
120°E
123°E
126°E
129°E

Figure S3.

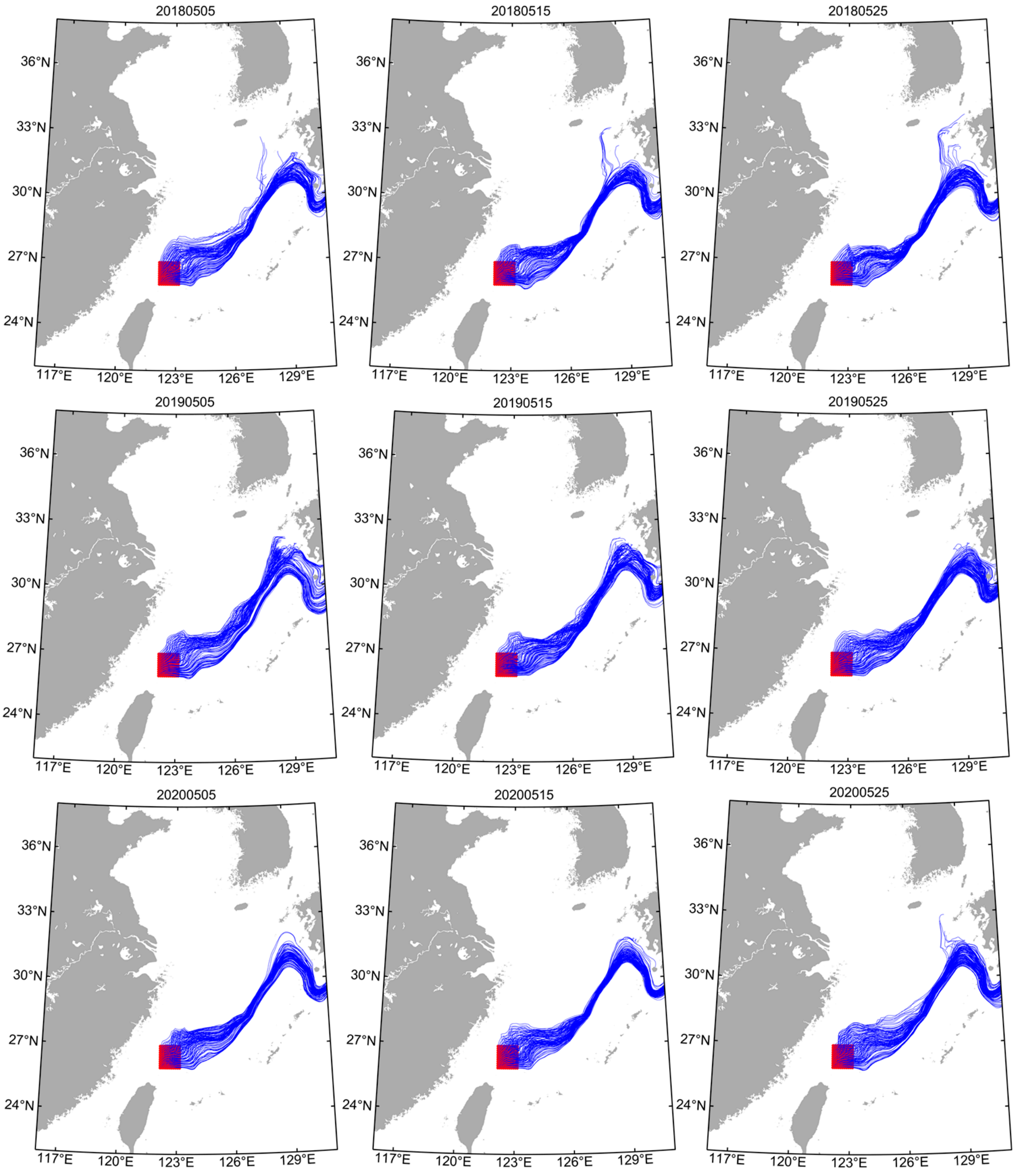
20180505
20180515
20180525
20190505
20190515
20190525
20200505
20200515
20200525
36°N
33°N
30°N
27°N
24°N
117°E
120°E
123°E
126°E
129°E

Figure S4.

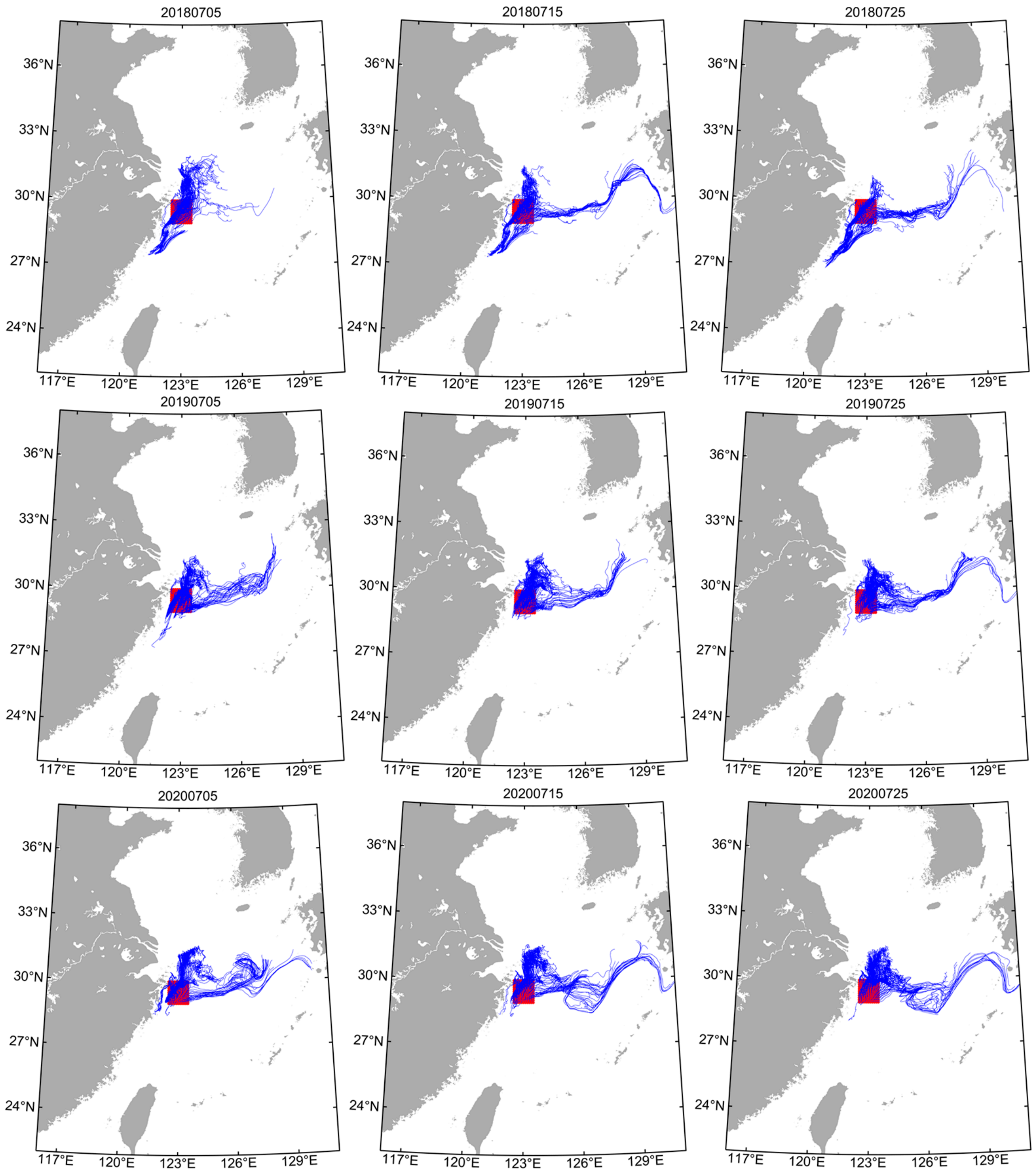
20180705
20180715
20180725
20190705
20190715
20190725
20200705
20200715
20200725
36°N
33°N
30°N
27°N
24°N
117°E
120°E
123°E
126°E
129°E

Figure S5.

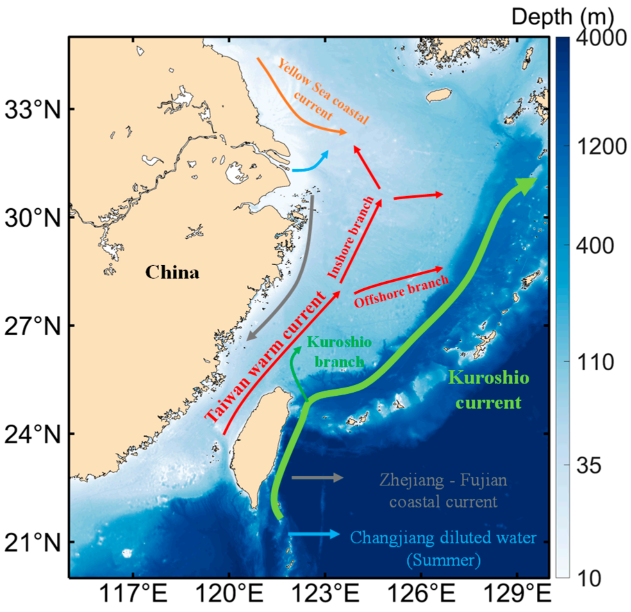
Depth (m)
4000
1200
400
110
35
10
33°N
30°N
27°N
24°N
21°N
117°E
120°E
123°E
126°E
129°E
Yellow Sea coastal current
China
Inshore branch
Offshore branch
Taiwan warm current
Kuroshio branch
Kuroshio current
Zhejiang - Fujian coastal current
Changjiang diluted water (Summer)

Figure S6.
